# Hfq Is a Critical Modulator of Pathogenicity of *Dickeya oryzae* in Rice Seeds and Potato Tubers

**DOI:** 10.3390/microorganisms10051031

**Published:** 2022-05-16

**Authors:** Zurong Shi, Qingwei Wang, Shunchang Wang, Chengrun Wang, Lian-Hui Zhang, Zhibin Liang

**Affiliations:** 1 Guangdong Laboratory for Lingnan Modern Agriculture, Guangdong Province Key Laboratory of Microbial Signals and Disease Control, Integrative Microbiology Research Centre, South China Agricultural University, Guangzhou 510642, China; shizurong207@163.com (Z.S.); wangqingwei265@126.com (Q.W.); 2 School of Biological Engineering, Huainan Normal University, Huainan 232038, China; wangrosen@163.com (S.W.); chengrunwang@163.com (C.W.); 3 Innovative Institute for Plant Health, Zhongkai University of Agriculture and Engineering, Guangzhou 510225, China

**Keywords:** zeamines, biofilm, hypersensitive response

## Abstract

The frequent outbreaks of soft-rot diseases caused by *Dickeya oryzae* have emerged as severe problems in plant production in recent years and urgently require the elucidation of the virulence mechanisms of *D. oryzae*. Here, we report that Hfq, a conserved RNA chaperone protein in bacteria, is involved in modulating a series of virulence-related traits and bacterial virulence in *D. oryzae* EC1. The findings show that the null mutation of the *hfq_EC1_* gene totally abolished the production of zeamine phytotoxins and protease, significantly attenuated the production of two other types of cell wall degrading enzymes, i.e., pectate lyase and cellulase, as well as attenuating swarming motility, biofilm formation, the development of hypersensitive response to *Nicotiana benthamiana*, and bacterial infections in rice seeds and potato tubers. QRT-PCR analysis and promoter reporter assay further indicated that Hfq_EC1_ regulates zeamine production via modulating the expression of the key zeamine biosynthesis (*zms*) cluster genes. Taken together, these findings highlight that the Hfq of *D. oryzae* is one of the key regulators in modulating the production of virulence determinants and bacterial virulence in rice seeds and potato tubers.

## 1. Introduction

*Dickeya* spp. are destructive plant pathogenic bacteria worldwide, which can cause soft-rot diseases in both monocotyledonous and dicotyledonous plants [1]. Rice foot rot disease, caused by one of the members of *Dickeya* spp., i.e., *D. oryzae*, has posed a great threat to not only rice production in China and some other Asian countries, including India, Indonesia, and Japan [2,3,4], but also to potato production in Australia [5]. Recent works unveiled the virulence determinants of *D. oryzae*, including phytotoxic zeamines, motility, cell wall–degrading enzymes (CWDEs), and biofilm formation [6,7,8]. Production of these determinants is regulated at different bacterial growth stages by the AHL quorum-sensing signal [2], putrescine signal [8], transcriptional regulators [9,10], the two-component signal transduction system [11,12], and the second messenger cyclic-di-GMP [13,14]. Until now, the contributions of small RNAs (sRNAs) and their chaperone proteins to the pathogenicity of *D. oryzae* are still unclear. 

Post-transcriptional regulation in bacteria is typically mediated by the base-pairing interactions between regulatory small RNAs (sRNAs) and their target mRNA transcripts, the outcome of which changes either translational efficiency, or mRNA stability, or both [15,16]. The interactions between sRNAs and their targets are often assisted by the RNA chaperone protein, i.e., Hfq. Hfq was firstly characterized in *Escherichia coli* as an essential host factor required for the replication of RNA bacteriophage Qbeta [17]. Subsequent studies unveiled that Hfq is a highly conserved, global sRNA chaperone in Gram-positive and -negative bacterial species [18,19], broadly involved in the regulation of bacterial physiology and metabolism, including membrane protein composition, stress tolerance, motility, and biofilm formation [20,21]. 

Previous works have been carried out to examine the role of Hfq in the growth of pathogenic bacteria. Mutation of *hfq* can impair the in vitro multiplication of bacterial pathogens belonging to *E. coli* [22], *Vibrio cholerae* [23], *Yersinia pseudotuberculosis* [24], and *Dickeya dadantii* [25] but has no effect on the growth of *Xanthomonas campestris* pv. *campestris* [26]. In addition, recent advances indicate that Hfq contributes to the pathogenicity of plant pathogens belonging to *Agrobacterium tumefaciens* [27], *Pantoea ananatis* [28], *Pectobacterium carotovorum* [29], *X. campestris* pv. *campestris* [26], and *Erwinia amylovora* [30]. However, Hfq is not implicated in the virulence of *Xanthomonas* *campestris* pv. *vesicatoria* and *Xanthomonas* *oryzae* pv. *oryzae* [31,32]. In *D. dadantii* 3937, Hfq was found to be essential for regulation of bacterial virulence. Inactivation of *hfq* strongly reduced bacterial motility and production of CWDEs, i.e., cellulose (Cel), pectate lyase (Pel), and protease (Prt), and compromised the capacity of *D. dadantii* 3937 to cause soft rot in chicory leaves [25]. 

Considering the great contributions of Hfq to bacterial growth, the production of virulence factors, and virulence in *D. dandatii*, we hypothesize that Hfq also plays significant roles in the virulence of *D*. *oryzae*. To elucidate the roles of Hfq in *D. oryzae*, in this study, we constructed an in-frame deletion mutant of *hfq* in *D. oryzae* EC1. Then, we assessed the influence of the inactivation of *hfq* on bacterial growth, the production of virulence-related traits, and bacterial virulence in rice seeds and potato tuber slices in *D. oryzae* EC1, steps which aimed to determine whether Hfq is an important modulator in the virulence of *D*. *oryzae* and to expand understanding of the roles of Hfq in different *Dickeya* species.

## 2. Materials and Methods

### 2.1. Bacterial Strains, Plasmids, and Cultural Conditions

The bacterial strains and plasmids used in this study are listed in Supplementary Table S1. *D. oryzae* EC1 and its derivatives were cultivated at 28 °C in minimal medium (MM), LS_5_ medium, and Luria–Bertani (LB) medium, as indicated [9]. The *E. coli* strains were grown at 37 °C in LB medium. Antibiotics were added to the medium at the following final concentrations when required: ampicillin, 100 µg/mL; streptomycin, 50 µg/mL; kanamycin, 50 µg/mL.

### 2.2. Construction of the Hfq_EC1_ Deletion Mutant and Complementation Strain

The method used for constructing the deletion mutant of *hfq* gene in *D. oryzae* EC1 (*hfq*_EC1_) and the corresponding complementation strain were previously described [8]. Briefly, fragments containing about a 500 bp upstream and downstream region of the *hfq*_EC1_ were amplified from the genomic DNA of wild-type strain EC1, respectively, and fused by overlapping extension PCR with the forward primer of the upstream fragment (Primer A-1, Supplementary Table S2) and the reverse primer of the downstream fragment (Primer A-4, Supplementary Table S2). The PCR product was purified, digested with restriction enzymes, and ligated to the vector pKNG101 digested with the same enzymes. The resultant construct was transformed into *E. coli* CC118 competent cells by heat shock at 42 °C and introduced into wild-type strain EC1 through triparental mating with the helper strain HB101 (pRK2013). The desired mutants were screened on MM agar plates containing 5% (wt/vol) sucrose and confirmed by PCR and DNA sequencing. For complementation, the coding sequence of *hfq*_EC1_ was amplified from the genomic DNA of wild-type strain EC1 with the primer pairs HB-A-F and HB-A-R (Supplementary Table S2). The PCR product was digested with restriction enzymes and introduced into pBBR1-MCS4 digested with the same enzymes. The desired complementation construct was introduced into the Δ*hfq_EC1_* by triparental mating and confirmed by PCR. The gene is expressed under the control of the *lac* promoter in pBBR1-MCS4.

### 2.3. Cell Wall–Degrading Enzyme Activities

The methods used for measuring the activity of Cel, Pel, and Prt were previously described, with minor modifications [13]. The compositions of media used for enzyme activity assays were as follows: Cel medium—carboxymethyl cellulose 1.0 g/L, Na_3_PO_4_ 3.8 g/L, agarose 8.0 g/L, pH 7.0; Pel medium—polygalacturonic acid 10 g/L, yeast extract 10 g/L, CaCl_2_ 0.1125 g/L, Tris-HCl 100 mM, agarose 8 g/L, pH 8.5; and Prt medium—the LB medium containing equal volume of 1% (wt/vol) skimmed milk. For semi-quantitative assays, wells of 5 mm were punched in plates. Twenty microliters of bacterial cell cultures (OD_600_ of 1.5) were added to the wells in plates with incubation at 28 °C. In Pel assay, the plates were treated with 1 N HCl after the incubation for 11 h. In Cel assay, the plates were developed by 0.1% (wt/vol) Congo Red staining and sequentially decolorized by 1 M NaCl. Halos around the wells became visible in Prt assay plates after 20 h without any further treatment.

### 2.4. Motility Assay and Biofilm Formation

Motility assay was performed by using a method described previously [8]. Briefly, swimming motility was elucidated in the semi-solid medium plate containing about 15 mL of semi-solid Bacto tryptone agar medium (Bacto tryptone 10 g/L, NaCl 5 g/L, and agar 3 g/L). An aliquot of 2 μL overnight bacterial culture was inoculated into 15 mL of semi-solid agar medium in the plates. The diameters of swimming motility were measured after the incubation at 28 °C for 24 h. In swarming motility assay, bacterial cells were inoculated in the middle of the plate, containing 15 mL of semi-solid agar medium (Tryptone 10 g/L, NaCl 5 g/L, and agar 4 g/L). The plates were incubated at 28 °C for 18 h, and the diameters of the swarming motility were measured. 

To quantify the biofilm formation, overnight bacterial culture was 1:100 diluted into the super optimal broth plus glycerol (SOBG) medium (Tryptone 20 g/L, yeast extract 5 g/L, MgSO_4_ 2.4 g/L, NaCl 0.5 g/L, KCl 0.186 g/L, and glycerol 2 g/L) in the 96-well plates. The plates were incubated at 28 °C with shaking at 150 rpm for 18 h. After the incubation, bacterial cell cultures were removed and an aliquot of 200 μL crystal violet (0.1% wt/vol) was added into each well for a 15 min staining. After the staining, the unbound crystal violet was removed and the wells were rinsed three times with water. The remaining crystal violet in each well was decolorized with 200 μL of 95% ethanol after dryness and quantified by measuring the absorbance at 570 nm. 

### 2.5. Zeamine Production Assay 

Zeamines are a family of structurally related phytotoxins required for the virulence of *D. oryzae* EC1 in rice seeds and potato tubers [6,7,33]. In addition, they are also potent antibiotics having broad activity against various organisms, including bacteria, fungi, and nematodes [33,34,35]. Zeamine production in the wild-type strain EC1 and the *hfq*_EC1_ deletion mutant were determined by measuring the antibiotic activity of zeamines against *E. coli* DH5α [7]. Briefly, bacterial cell cultures grown overnight in LS_5_ medium to an OD_600_ of 1.5 were filtered. An aliquot of 30 μL cell-free supernatants was added into the wells in bioassay plates, in which the 15 mL LB agar medium was overlaid with 5 mL of 1% agarose containing about 1.0 × 10^8^ cells of *E. coli* DH5α. The plates were incubated at 37 °C for 18 h to measure the radius of the visible clear zone surrounding the wells in order to determine the production of zeamines in *D. oryzae* EC1 and *hfq*_EC1_ mutant.

### 2.6. RNA Extraction and Quantitative Real-Time Reverse-Transcription PCR (qRT-PCR) Analysis

The wild-type strain EC1 and *hfq*_EC1_ mutant were grown in LS_5_ medium to an OD_600_ about 1.5. The RNA samples were prepared using the SV total RNA isolated system kit (Promega, Beijing, China) and further purified using the RNA clean kit (Qiagen, Hilden, Germany). The cDNA synthesis was performed by using StarScript II first-strand cDNA synthesis Mixing followed the manufacturer’s instructions (GenStar Biosolutions, Beijing, China). The qRT-PCR analysis was conducted on a Quantstudio 6 Flex system using PowerUp SYBR green master mix (Thermo Fisher Scientific, Waltham, MA, USA) with the primers listed in Supplementary Table S2 and followed cycle profile: 1 cycle at 50 °C for 2 min and 95 °C for 2 min, followed by 40 cycles at 95 °C for 5 s and 60 °C for 30 s. Data were analyzed using the 2^−ΔΔCT^ method, as previously described [36].

### 2.7. Pathogenicity Assay on Potato Tuber Slices

Pathogenicity assay on potato tubers was performed by using methods described previously [7]. Briefly, potato tubers were sliced evenly to 5 mm in thickness after washing and surface disinfestation. An aliquot of 2 μL of bacterial cell cultures at an OD_600_ of 1.5 was added to the center of each sliced potato tuber. The potato tuber slices were then incubated at 28 °C and observed regularly for symptom development. 

### 2.8. Rice Seed Germination Assay

The rice seed germination assay was conducted as previously described [2]. Briefly, overnight bacterial cultures were diluted in 10-fold series and the CFU (colony-forming unit) of each dilution was determined by using the plate counting assay. Thirty seeds of rice variety CO39 were added to 9 mL of bacterial dilution and incubated at room temperature (25 °C) for 5 h. After the incubation, the rice seeds were washed three times with sterilized water and subsequently transferred onto the moistened filter papers in petri dishes for incubation at 28 °C with a 16 h light and 8 h dark cycle. Rice seeds incubated with the same amount of sterilized water were considered as the control. The sterilized water was added to the filter papers during incubation when necessary. After incubation for 7 d, the rate of seed germination was determined. 

### 2.9. Hypersensitive Response Assay

The hypersensitive responses (HR) of *D. oryzae* EC1 and its derivatives were tested on the nonhost plant *Nicotiana benthamiana*. The upper surface of leaf was inoculated by infiltrating approximately 5 μL of bacterial cell cultures (OD_600_ = 0.05, 2.0 × 10^4^ CFU/mL) using a 1 mL blunt-end plastic syringe. The inoculated plants were incubated in a greenhouse with a 12 h day-and-night cycle at 28 °C. HR symptoms were photographed 24 h post-inoculation. At least three plants were inoculated in each experiment.

### 2.10. Statistical Analysis 

Experiments were individually performed at least three times with three replicates each time. Statistical comparison was performed by using Student’s *t* test in GraphPad Prism 5.0 software (GraphPad, La Jolla, CA, USA). A *p* value of less than 0.05 was considered significant.

## 3. Results

### 3.1. Identification of Hfq in D. oryzae EC1

**Figure 1:**
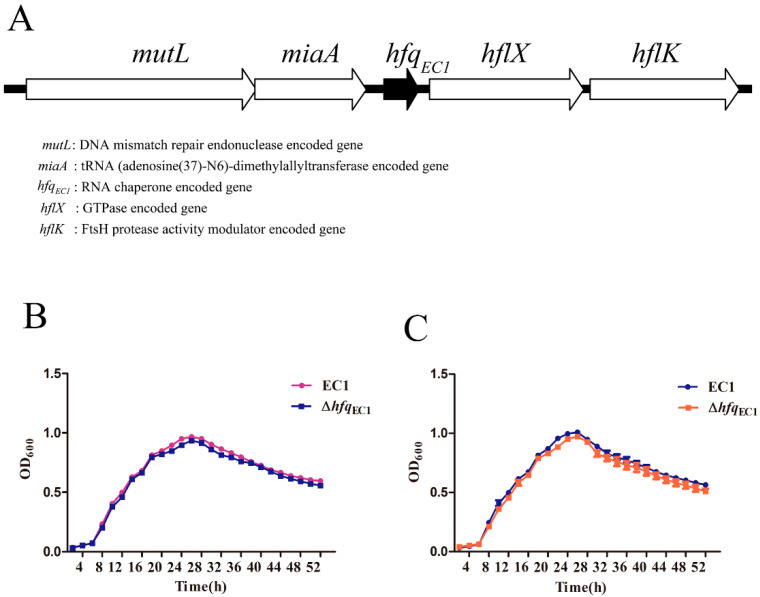
Gene organization of *hfq_EC1_* and its influence on bacterial growth in *Dickeya oryzae*. (**A**) Gene organization of *hfq_EC1_*. Growth patterns of the wild-type strain EC1 and the *hfq_EC1_* deletion mutant were measured in Luria–Bertani (LB) medium (**B**) and minimal medium (MM) (**C**).

Homology blast and sequence alignment unveiled that a putative *hfq* gene, named *hfq*_EC1_, exhibits 82% sequence similarity at the amino acid level (NCBI accession no. WP_012886271) compared with the *hfq* in *E. coli* (NCBI accession no. ACE63256.1) and is present in *D. oryzae* EC1 (Figure 1A). To determine the role of *hfq*_EC1_ in the growth of *D. oryzae* EC1, we compared the growth patterns of wild-type strain EC1 and Δ*hfq*_EC1_ in rich medium (LB) and the minimal medium (MM), respectively. The results showed that the *hfq*_EC1_ mutant had a comparable growth pattern to the wild-type strain EC1 in both rich and minimal media (Figure 1B,C), which suggests *hfq*_EC1_ is not required for the growth of *D. oryzae* EC1 in vitro.

### 3.2. Deletion of Hfq_EC1_ Decreases the Production of Extracellular Degrading Enzymes in D. oryzae EC1

**Figure 2:**
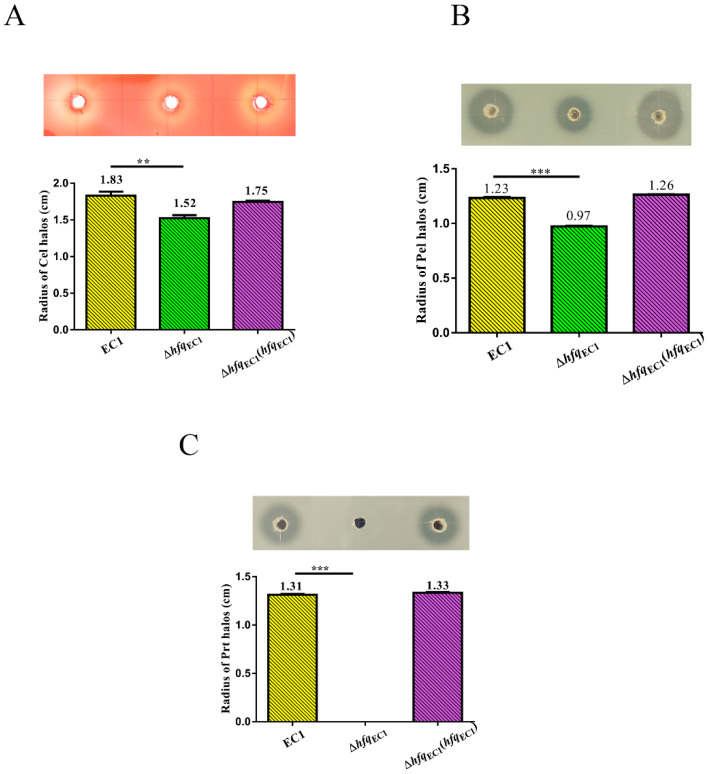
Hfq_EC1_ regulates the production of cell wall–degrading enzymes. The wild-type strain EC1 of *Dickeya oryzae* and its derivatives were cultured in Luria–Bertani (LB) medium to an OD_600_ of 1.5. Production of Cel (**A**), Pel (**B**), and Prt (**C**) was measured in the media with their substrates and the corresponding methods. For semi-quantification of enzyme production, the radius of each halo was measured. Experiments were individually performed at least three times in triplicate. The data shown are the means ± SE (*n* = 3). ** *p* < 0.05; *** *p* < 0.01.

To elucidate whether *hfq*_EC1_ is responsible for the production of CWDEs in *D*. *oryzae* EC1, the production of CWDEs in wild-type strain EC1 and *hfq*_EC1_ mutant were compared with the agar plates with substrates of Pel, Cel, and Prt. The results showed that the production of Cel and Pel in the *hfq*_EC1_ mutant decreased by 17% and 21%, respectively, compared with those in wild-type strain EC1 (Figure 2A,B). Notably, inactivation of *hfq*_EC1_ totally abolished the production of Prt in *D. oryzae* EC1 (Figure 2C). The complementation analysis showed that *in trans* expression of *hfq*_EC1_ could restore the production of all these three types of CWDEs (Figure 2A–C). These results indicate that the Hfq_EC1_ is required for the production of CWDEs in *D. oryzae* EC1, especially Prt.

### 3.3. Hfq_EC1_ Contributes to Swarming Motility and Biofilm Formation in D. oryzae EC1

**Figure 3:**
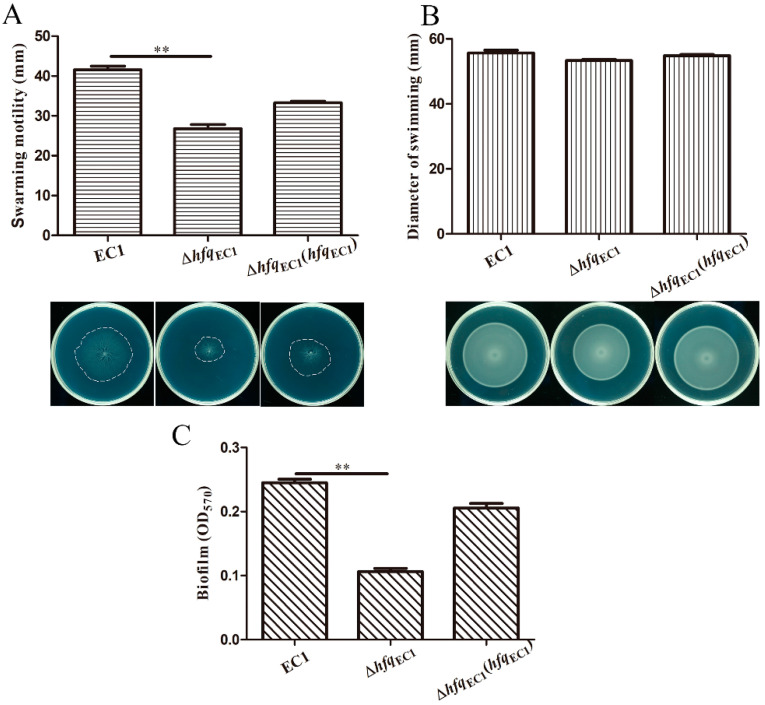
Disruption of *hfq_EC1_* decreases bacterial swarming motility and biofilm formation. (**A**) Swarming motility of *Dickeya oryzae* EC1 and its derivatives. The diameters were measured after incubation at 28 °C for 18 h. (**B**) Swimming motility of *D. oryzae* EC1 and its derivatives. The plates were incubated at 28 °C for 24 h before photography. (**C**) Biofilm formation of *D. oryzae* EC1 and its derivatives. Bacterial strains were grown in super optimal broth plus glycerol (SOBG) medium at 28 °C with shaking for 18 h. Experiments were individually performed at least three times in triplicate. The data shown are the means ± SE (*n* = 3). ** *p* < 0.05.

To elucidate whether Hfq_EC1_ play key roles in motility and biofilm formation in *D. oryzae* EC1, the motility and biofilm-forming capacity of wild-type strain EC1 and the *hfq*_EC1_ deletion mutant were compared. The result showed that the Δ*hfq*_EC1_ displayed a comparable swimming motility but a decreased swarming motility compared with the wild-type strain EC1 (Figure 3A,B). Consistently, the crystal violet staining assay performed in the 96-well plates revealed that the biofilm formation in the *hfq*_EC1_ mutant represented a significant decrease compared with that in wild-type strain EC1 (Figure 3C). All these results indicate that Hfq_EC1_ contributes to the regulation of swarming motility and biofilm formation in *D. oryzae* EC1.

### 3.4. Hfq_EC1_ Regulates Zeamine Production through Modulating the Expression of Key Zms Cluster Genes 

**Figure 4:**
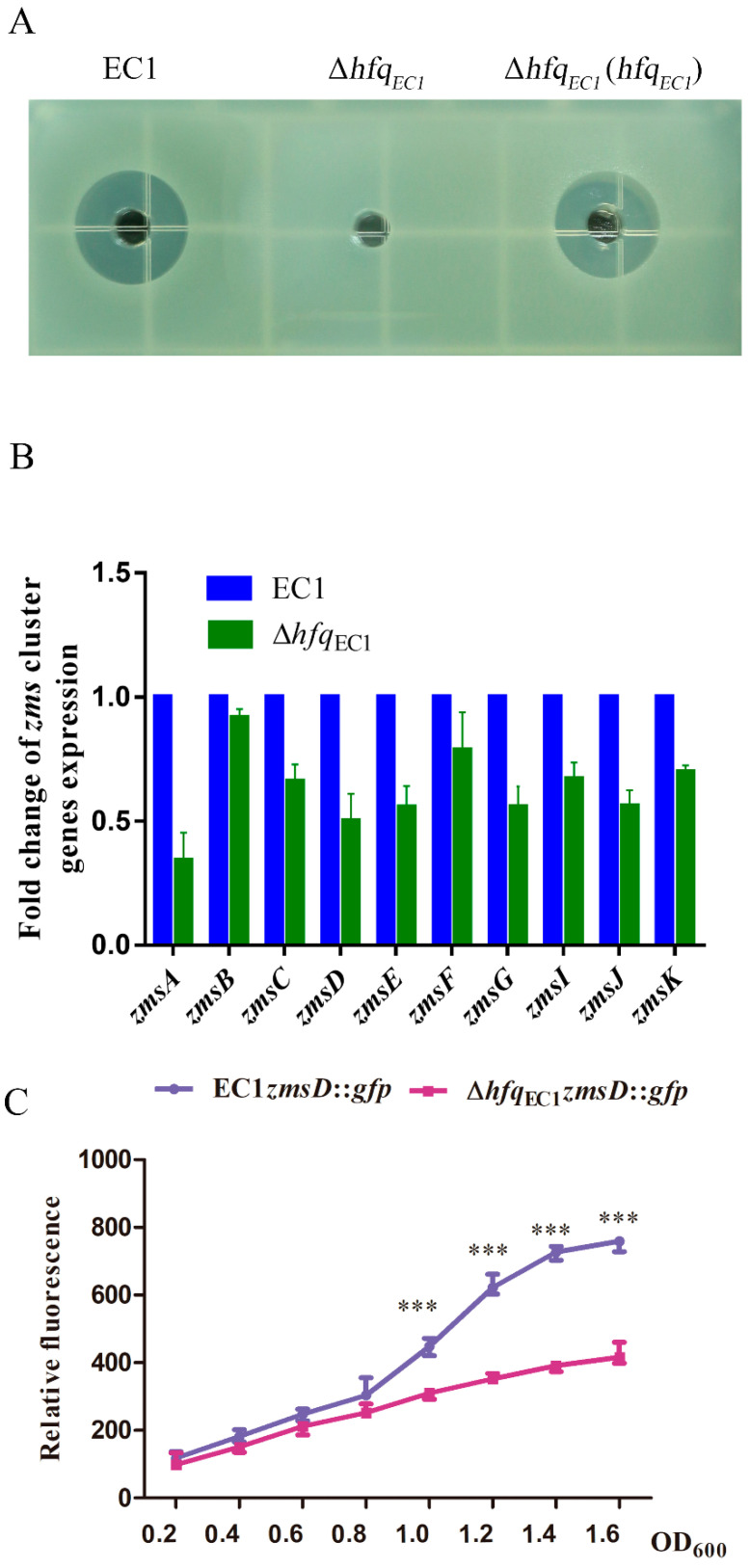
Expression of the *zms* cluster genes in *Dickeya oryzae* EC1 is regulated by Hfq_EC1_. (**A**) Zeamine production assay. The filter-sterilized bacterial supernatants of *D. oryzae* EC1 and its derivatives were added to the wells in the bioassay plates, respectively. The bioassay plates were incubated at 37 °C for 24 h before photography. Experiments were repeated at least three times in triplicates. (**B**) QRT-PCR assay reveals the transcriptional regulation of key *zms* cluster genes by Hfq_EC1_. (**C**) GFP promoter reporter fusion assay shows the regulation of *zmsD* gene by Hfq_EC1_. Experiments were individually performed at least three times in triplicates. *** *p* < 0.01.

To elucidate whether Hfq_EC1_ is required for the production of zeamines in *D. oryzae* EC1, zeamine assays were performed on wild-type strain EC1 and the *hfq*_EC1_ mutant. Intriguingly, the result showed that inactivation of *hfq*_EC1_ resulted in a complete loss of zeamine production in *D. oryzae* EC1. Conversely, *in trans* expression of *hfq*_EC1_ could restore the zeamine production of the *hfq*_EC1_ mutant (Figure 4A). This finding shows that Hfq_EC1_ plays a key role in modulating zeamine production in *D. oryzae* EC1. To elucidate whether the zeamine production conferred by Hfq_EC1_ relies on the transcriptional regulation of key *zms* cluster genes [37], the expression of *zmsA*–*G* and *zmsI*–*K* was determined by qRT-PCR assay when bacterial strains were cultured in the medium optimized for zeamine production (LS_5_ medium). The result indicated that expression of the key *zms* cluster genes in *hfq_EC1_* mutant, especially the *zmsA*, was decreased to different degrees compared with that in the wild-type strain EC1 at an OD_600_ of 1.5, where zeamines are largely produced by *D. oryzae* EC1 [38] (Figure 4B). In addition, the GFP promoter reporter fusion assay further showed that expression of *zmsD* in wild-type strain EC1 was continuously increased and higher than that in the *hfq*_EC1_ mutant at an OD_600_ from 0.8 to 1.6 in LS_5_ medium (Figure 4C). These results indicate that Hfq_EC1_ controls zeamine production mainly through regulating the expression of key *zms* cluster genes. 

### 3.5. Hfq_EC1_ Is Required for the Virulence of D. oryzae EC1

**Figure 5:**
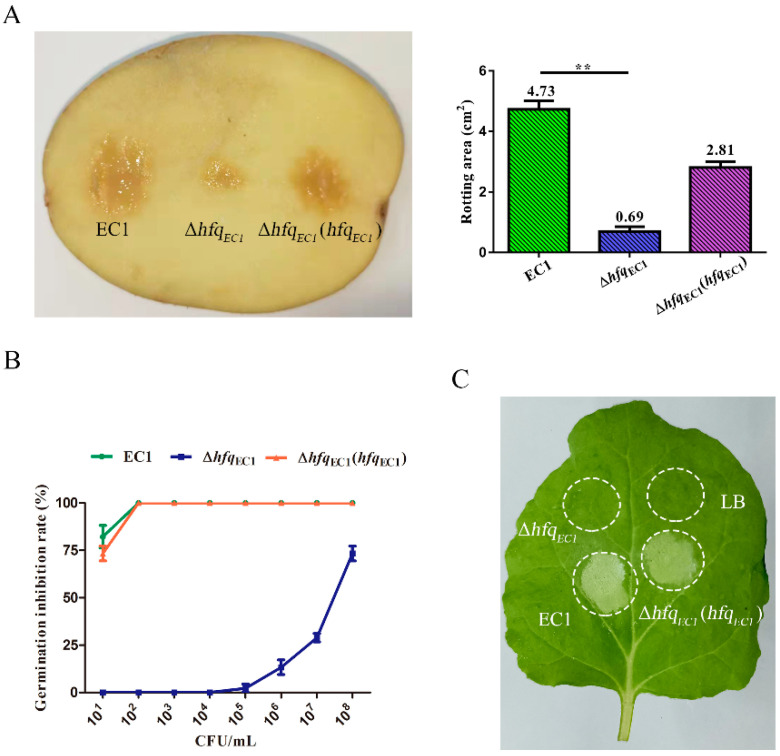
Hfq_EC1_ is required for the virulence and hypersensitive response (HR) in *Dickeya oryzae* EC1. (**A**) Disease symptoms (left) and the rotting areas (right) on potato tubers inoculated with wild-type strain EC1, *hfq_EC1_* mutant, and complementation strain of *hfq_EC1_* mutant. Photograph was taken 2 d after inoculation. The numbers at the top of each column show the average rotting area ± SD from three repeats. ** *p* < 0.01. (**B**) Analysis of the capacity of *D*. *oryzae* EC1 in inhibiting rice seed germination. Rice seeds were treated with different bacterial dilutions as indicated and incubated at 28 °C for 7 days. Experiments were individually performed at least three times in triplicate. (**C**) The hypersensitive response induced by wild-type strain EC1, *hfq_EC1_* mutant, and complementation strain of *hfq_EC1_* mutant on leaves of *Nicotiana benthamiana*. An aliquot of 5 μL of bacterial cell cultures (OD_600_ = 0.05, 2.0 × 10^4^ CFU/mL) was inoculated. The photograph was taken 24 h after infiltration.

To determine whether Hfq_EC1_ is involved in the regulation of the pathogenicity of *D. oryzae* EC1, we compared the infections of wild-type strain EC1 and the *hfq*_EC1_ mutant in rice seeds and potato tuber slices. The results showed that potato tuber slices inoculated with the *hfq*_EC1_ mutant had substantially reduced rotting areas compared with those inoculated with the wild-type strain EC1 at 48 h post-inoculation (Figure 5A). Similarly, the wild-type strain EC1 was much more virulent than the *hfq*_EC1_ mutant on rice seed germination, showing about an 82% inhibition rate when rice seeds were treated with 10 bacterial cells per mL and total inhibition at 100 bacterial cells per mL. Compared with the wild-type strain EC1, the *hfq*_EC1_ mutant was unable to inhibit rice seed germination at a concentration range from 10 to 10,000 bacterial cells per mL, only showing about a 73% inhibition rate when the bacterial inoculation was increased to 10^8^ cells per mL (Figure 5B). Conversely, *in trans* expression of *hfq*_EC1_ in *hfq*_EC1_ mutant restored bacterial infective capacity in rice seed germination and potato tuber slices (Figure 5A,B). Taken together, these findings indicate that Hfq_EC1_ is required for the virulence of *D. oryzae* EC1 in both monocotyledonous and dicotyledonous plants. 

To determine whether Hfq_EC1_ is required for triggering hypersensitive response (HR) on nonhost plants in *D. oryzae* EC1, the HR symptoms on *N. benthamiana* leaves developed by wild-type strain EC1 and the *hfq*_EC1_ mutant were compared. The results showed that HR lesion developed by the *hfq*_EC1_ mutant was defective in size compared with that developed by wild-type strain EC1 after 24 h inoculation (Figure 5C), suggesting that Hfq_EC1_ is essential for *D. oryzae* EC1 to trigger HR in nonhost plant *N. benthamiana*.

## 4. Discussion

The Hfq proteins are conserved and essential regulators for regulating the production of divergent virulence factors in a large number of bacterial pathogens. In this study, we systematically elucidated the roles of Hfq_EC1_ in the production of virulence factors and bacterial virulence in *D*. *oryzae* EC1. Inactivation of the *hfq_EC1_* totally abolished the production of Prt and zeamines in *D*. *oryzae* EC1 (Figure 2C and Figure 4A) and dramatically reduced the pathogenicity of *D*. *oryzae* EC1 in potato tubers and rice seeds (Figure 5A,B). QRT-PCR and promoter reporter assay further showed that Hfq_EC1_ modulates the production of zeamines through transcriptional regulation of key *zms* cluster genes. The transcriptional regulation of *zms* cluster genes by Hfq_EC1_ may suggest cooperation between Hfq_EC1_ and other transcriptional regulatory mechanisms through sRNA-based post-transcription regulation in *D*. *oryzae* EC1.

Previous studies indicated that inactivation of *hfq* could cause reduced bacterial growth rates in a large proportion of bacterial species, including *D. dadantii*, *P. carotovorum*, *A. tumefaciens*, *P. ananatis*, and *E. amylovora* [25,27,28,29,30]. However, despite the relatively close taxonomic relationship between *D. dadantii* and *D*. *oryzae*, similar to bacterial strain belong to *X. campestris* pv. *campestris* [26], we found that the growth rates of *hfq_EC1_* mutant were comparable to those of the wild-type strain EC1 in the selected culture conditions (Figure 1B,C), which suggests the defective phenotype of the *hfq_EC1_* mutant is not related to the proposed involvement of Hfq_EC1_ in the growth of *D*. *oryzae* EC1, and Hfq plays differential roles in different *Dickeya* species.

Zeamines are crucial to the infection of *D. oryzae* EC1 in rice seeds [6,7]. In this study, we found the capacity of *D*. *oryzae* EC1 for inhibiting rice seed germination was largely impaired at each inoculation concentration after inactivation of *hfq_EC1_* (Figure 5B). This resulted from the contribution of Hfq_EC1_ to the production of zeamines (Figure 4A). The role of Hfq in regulation of zeamine production was also reported in a biocontrol bacterial isolate, i.e., *Serratia plymuthica* A153 [34]. Inactivation of *hfq* in *S*. *plymuthica* A153 totally abolished the zeamine production. All these factors suggest the conserved regulatory role of Hfq in zeamine production across different bacterial species. Hfq is closely associated with the RsmA/RsmB signaling pathway in the regulation of biofilm formation in *E. coli* [39] and the production of CWDEs in *D. dadantii* 3937 [40]. In *D. oryzae* EC1, our recent work unveiled that the regulons of *rmsB*, i.e., TzpS-TzpA, which are homologous to GacS-GacA, are also implicated in the production of zeamines through transcriptional regulation of *zms* cluster genes [12]. In this study, we found that Hfq_EC1_ regulated the expression of *zms* cluster genes at transcriptional level. The proposed cross-talk among TzpS-TzpA, RsmA/RsmB, and Hfq_EC1_ in the transcriptional regulation of *zms* gene expression is intriguing and requires further elucidation.

The CWDEs are essential for plant pathogens to develop soft-rot symptoms in plants. The regulatory roles of Hfq proteins on the production of CWDEs were documented in soft-rot plant pathogens belonging to *Dickeya* and *Pectobacterium* [25,29]. In this work, we showed that production of Prt was totally abolished in the *hfq_EC1_* deletion mutant (Figure 2C). Moreover, significant reductions on the production of Pel and Cel were also noticed in the *hfq_EC1_* mutant compared with wild-type strain EC1 (Figure 2A,B). Consistent with these findings, the null mutation of *hfq_EC1_* attenuated the rotting areas on potato tubers (Figure 5A). Compared with the *hfq* in well-studied *D. dadantii* 3937, which plays a major role in the production of Pel [25], we found that *hfq_EC1_* was largely involved in the production of Prt but not Pel (Figure 2B,C), which suggests Hfq proteins confer divergent regulatory networks for the production of CWDEs in *D. dadantii* and *D*. *oryzae*. 

The HR is a phenotype of programmed cell death that bacterial pathogens can induce through the type-III secretion system (T3SS) in nonhost plants [41,42]. Although the Hfq proteins in plant pathogens are frequently associated with the production of CWDEs, motility, and biofilm formation, they did not commonly contribute to the induction of HR in plant pathogens. The association between Hfq and HR developed by plant pathogens are so far reported only in a few bacterial species, including *E. amylovora* and *D. dadantii*. In *E. amylovora*, Hfq regulates the translocation and secretion of the effector DspE [30]. In *D. dadantii* 3937, Hfq modulates the expression of T3SS though positively regulating the expression of *rsmB* at the post-transcriptional level [40]. In this study, we found that the null mutation of *hfq_EC1_* not only dramatically attenuated the virulence of *D. oryzae* EC1 in host plants, i.e., potato and rice, but also abolished the development of HR symptoms in nonhost plant *N. benthamiana* (Figure 4), which suggests a potential link between Hfq and expression of the type-III secretion system in *D. oryzae* EC1.

## 5. Conclusions

In summary, this study unveiled the key role of the RNA chaperone protein Hfq_EC1_ in the production of virulence-related traits, particularly Prt and zeamine phytotoxins, both of which are key virulence factors required for virulence of *D. oryzae* EC1 in rice seeds and potato tubers. In addition, this study highlights the divergence of regulatory networks mediated by Hfq proteins for bacterial growth and production of CWDEs in different *Dickeya* species. In a further study, it would be intriguing to elucidate the Hfq_EC1_-dependent sRNA regulatory network and its cross-link with the previously determined transcriptional regulatory mechanisms required for zeamine production in *D. oryzae* EC1. 

## Data Availability

The genomic sequence of *D. oryzae* EC1 in NCBI is accessible with No. NZ_CP006929.1.

## Supplementary Materials

The following supporting information can be downloaded at: https://www.mdpi.com/article/10.3390/microorganisms10051031/s1, Table S1. Strains and plasmids used in this study. Table S2. Primers used in this study.
