# Supplementary material for: Hfq Is a Critical Modulator of Pathogenicity of Dickeya oryzae in Rice Seeds and Potato Tubers
Source: Microorganisms. 2022 May 16;10(5):1031. doi: 10.3390/microorganisms10051031 (PMC9144144; doi:10.3390/microorganisms10051031)
Supplement: Supplementary file 1 [file microorganisms-10-01031-s001.zip › microorganisms-1715207-supplementary.pdf]

Supplementary Table S1. Strains and plasmids used in this study

Strains or plasmids	Relevant phenotypes and characteristics[a]	Source or reference
Dickeya oryzae		
EC1	Wild-type rice rot pathogen of *Dickeya oryzae* , Pmb^r	[1]
Δ*hfq*$_{EC1}$	*hfq*$_{EC1}$ deletion mutant derived from EC1	This study
Δ*hfq*$_{EC1}$ (*hfq*$_{EC1}$)	The complemented strain of Δ*hfq*$_{EC1}$, Amp^r	This study
EC1 *zmsD*::*gfp*	EC1 with a *gfp* transcriptional fusion in the coding sequence of *zmsD*	This study
Δ*hfq*$_{EC1}$ *zmsD*::*gfp*	Δ*hfq*$_{EC1}$ with a *gfp* transcriptional fusion in the coding sequence of *zmsD*	This study
Escherichia coli		
CC118	Host for plasmids constructed of pKNG101	Lab collection
DH5α	Host for plasmids constructed of pBBRI-MCS4	Lab collection
HB101(pRK2013)	*Thr leu thi recA hsdR hsdM pro,* Km^r	Lab collection
**Plasmids**		
pKNG101	Knockout vector, Str^r	Lab collection
pKNG101-*hfq*$_{EC1}$	pKNG101 containing in-frame deleted fragment of *hfq*$_{EC1}$, Str^r	This study
pKNG101-*zmsD-gfp*	pKNG101 containing in-frame deleted fragment of *zmsD* and ORF of *gfp*, Str^r	This study
pBBRI-MCS4	Multicopy expression vector, Amp^r	Lab collection
pBBRI- *hfq*$_{EC1}$	pBBRI-MCS4 containing the coding sequence of *hfq*$_{EC1}$ at the downstream of *lac* promoter, Amp^r	This study

[a]Pmb^r, Amp^r, Km^r, or Str^r: Resistance to polymyxin B, ampicillin, kanamycin, or streptomycin, respectively.

Reference

1. Hussain, M.B.B.M.; Zhang, H.B.; Xu, J.L.; Liu, Q.; Jiang, Z.; Zhang, L.H. The Acyl-Homoserine Lactone-Type Quorum-Sensing System Modulates Cell Motility and Virulence of *Erwinia chrysanthemi* pv. zeae. *J. Bacteriol.* **2008**, *190*, 1045–1053, doi:10.1128/JB.01472-07.

Supplementary Table S2. Primers used in this study

Primers	Description	Sequences
A-1	Forward primer for upstream of hfq_{EC1}	5’-CGGGATCCAGAATCAACCCAGTGCGGTC-3’
A-2	Reverse primer for upstream of hfq_{EC1}	5’-GCTAAGGGGCAATCTTTGCACGCGTCCTTACCAGTTTACCAC-3’
A-3	Forward primer for downstream of hfq_{EC1}	5’-GTGGTAAACTGGTAAGGACGCGTGCAAAGATTGCCCCTTAGC-3’
A-4	Reverse primer for downstream of hfq_{EC1}	5’-GGGGTACCCAAAGACATCCGGTGAAGCG-3’
HB-A-F	Forward primer for the coding sequence of hfq_{EC1}	5’-CCCAAGCTTCGTTACGCACGGTGATTCAG-3’
HB-A-R	Reverse primer for the coding sequence of hfq_{EC1}	5’-CGGGATCCGGCGGGAAAAGTGCTGATTT-3’
zmsA-F	Forward primer for qPCR of gene *zmsA*	5’-ATCGCAGATATCCGCAGTGG-3’
zmsA-R	Reverse primer for qPCR of gene *zmsA*	5’-CGTACCGTAGCCTGTGACTC-3’
zmsB-F	Forward primer for qPCR of gene *zmsB*	5’-CGCCGTTTAAGGCGATTGAG-3’
zmsB-R	Reverse primer for qPCR of gene *zmsB*	5’-GGTGATCCCACAGGACGTTT-3’
zmsC-F	Forward primer for qPCR of gene *zmsC*	5’-CGTCGGGTCAGTGATATCGG-3’
zmsC-R	Reverse primer for qPCR of gene *zmsC*	5’-CATCAGGTGTGCAGTGTTGC-3’
zmsD-F	Forward primer for qPCR of gene *zmsD*	5’-AGCAGGTGGATCCGCTTATG-3’
zmsD-R	Reverse primer for qPCR of gene *zmsD*	5’-GGGCTACCGCAGTAACACTT-3’
zmsE-F	Forward primer for qPCR of gene *zmsE*	5’-ACAGTGCTAGTGGGCGTTAC-3’
zmsE-R	Reverse primer for qPCR of gene *zmsE*	5’-AACGGAACGTCAACCCAGTT-3’
zmsF-F	Forward primer for qPCR of gene *zmsF*	5’-TGTTAAAGCAGTGTCGGGGT-3’
zmsF-R	Reverse primer for qPCR of gene *zmsF*	5’-ATGGCCTTCCATCTGTTCCG-3’
zmsG-F	Forward primer for qPCR of gene *zmsG*	5’-CAGCAATTGTTGGCGTGGAA-3’
zmsG-R	Reverse primer for qPCR of gene *zmsG*	5’-CTCTATCGCCACCAGCTCAG-3’
zmsI-F	Forward primer for qPCR of gene *zmsI*	5’-GCGCGACGATACCAGTTTTC-3’
zmsI-R	Reverse primer for qPCR of gene *zmsI*	5’-ACACCAGTCAATGCTGCGTA-3’
zmsJ-F	Forward primer for qPCR of gene *zmsJ*	5’-CATCAGACTCGCTGCCGTAT-3’
zmsJ-R	Reverse primer for qPCR of gene *zmsJ*	5’-CCTTTCGGCGTCTCTGTTCT-3’
zmsK-F	Forward primer for qPCR of gene *zmsK*	5’-GTACATCAGATGAGCGGCGA-3’
zmsK-R	Reverse primer for qPCR of gene *zmsK*	5’-ATCCATCGGCACAGGCATAG-3’
gfp-F	Forward primer for coding sequence of *gfp*	5’-atgagccaacaagtcgctgtCCGCTGTCCTTGACTCCACTT-3’
gfp-R	Reverse primer for coding sequence of *gfp*	5’-ataacgatcccacccgcatcGCGCCCCGGCAAATTCCTG-3’
D-1	Forward primer for upstream of *zmsD*	5’-cccctgcaggtcgacggatccTGTATACCCGTGGCAAGGTGA-3’
D-2	Reverse primer for upstream of *zmsD*	5’-ACAGCGACTTGTTGGCTCATG-3’
D-3	Forward primer for downstream of *zmsD*	5’-GATGCGGGTGGGATCGTT-3’
D-4	Reverse primer for downstream of *zmsD*	5’-gctgcttctacttatggtaccAATACTCCCACCGAGGCAGAA-3’
